# Safety, pharmacokinetics and pharmacodynamics of a topical SYK inhibitor in cutaneous lupus erythematosus: A double‐blind Phase Ib study

**DOI:** 10.1111/exd.14253

**Published:** 2020-12-17

**Authors:** Alex Walker, Lars Erwig, Katie Foster, Katherine Nevin, Joerg Wenzel, Margitta Worm, Nicola Williams, Nirav Ratia, Bao Hoang, Tanja Schneider‐Merck, Sophie Gisbert, Heike Carnarius, Marion Dickson

**Affiliations:** ^1^ GSK Stevenage UK; ^2^ University Hospital of Bonn Bonn Germany; ^3^ Division of Allergy and Immunology Department of Dermatology, Venerology and Allergy Charité Berlin Germany; ^4^ GSK Collegeville PA USA; ^5^ GSK Hamburg Germany; ^6^ GSK Uxbridge UK

**Keywords:** cutaneous lupus erythematosus, interferons, pharmacology, safety, SYK Kinase

## Abstract

The immunoregulator spleen tyrosine kinase (SYK) is upregulated in cutaneous lupus erythematosus (CLE). This double‐blind, multicentre, Phase Ib study evaluated the safety, tolerability, pharmacokinetics, pharmacodynamics and clinical efficacy of the selective SYK inhibitor GSK2646264 in active CLE lesions. Two lesions from each participant (*n* = 11) were each randomized to topical application of 1% (w/w) GSK2646264 or placebo for 28 days; all participants received GSK2646264 and placebo. The primary endpoint was safety and tolerability of GSK2646264, assessed by adverse event incidence and a skin tolerability test. Secondary endpoints included change from baseline in clinical activity and mRNA expression of interferon‐related genes in skin biopsies. Levels of several immune cell markers were evaluated over time. Eight (73%) participants experienced ≥ 1 adverse event (all mild in intensity), and maximal dermal response was similar for GSK2646264 and placebo. The expression of several interferon‐related genes, including CXCL10 and OAS1, showed modest decreases from baseline after 28 days of treatment with GSK2646264 compared with placebo. Similar findings were observed for CD3 + T cell and CD11c + dendritic cell levels; however, overall clinical activity remained unchanged with GSK2646264 vs. placebo. Further studies are warranted to assess SYK inhibitors as potential treatment for CLE.

## 1 BACKGROUND

Cutaneous lupus erythematosus (CLE) is a heterogenous autoimmune skin disease with several subtypes, including acute, subacute and chronic.^1^, ^2^ Studies have demonstrated a substantial burden from CLE on healthcare systems and patient lives, highlighting an unmet treatment need.^3^, ^4^, ^5^ Consistent with this, patients have expressed a desire for disease‐modifying therapies that alleviate CLE symptoms and allow them to reduce the number of pills taken.^3^

Inflammation associated with cutaneous lupus lesions is likely driven through activation of the interferon (IFN) pathway and potentially mediated by spleen tyrosine kinase (SYK), suggesting that SYK is a putative target for treating CLE (Appendix S1).^6^, ^7^ The novel small molecule SYK inhibitor GSK2646264 has good potency, selectivity and skin permeability^8^ and has recently shown encouraging preclinical results as a topical treatment for skin mast cell diseases.^9^

## 2 QUESTIONS ADDRESSED

This study examined the safety, tolerability, pharmacokinetics, pharmacodynamics and clinical effects of repeat topical applications of GSK2646264 vs. placebo in participants with CLE lesions.

## 3 EXPERIMENTAL DESIGN

This double‐blind (sponsor‐unblinded) Phase Ib (GSK study number: 204860; ClinicalTrials.gov: NCT02927457) study was conducted across five centres in Germany between 13 January 2017 and 12 June 2018. Eleven participants with ≥2 active lesions were randomized into the “active lesion cohort” (6 chronic CLE, 5 subacute CLE) (Figure S1). A separate enrolment to a “photoprovocation cohort” was planned for participants with 0 or 1 active lesions to study the effect of GSK2646264 on photoprovocation‐induced, non‐scarred, non‐chronic lesions; however, no participants were randomized to this cohort due to feasibility of recruitment (Appendix S2). This report focuses on the active lesion cohort only.

Key inclusion criteria included were as follows: 18–70 years of age; histologically confirmed subacute or chronic CLE; free from scarring, skin markings or wounds in the areas to be treated; and no extended direct sunlight and tanning products on the areas to be treated. The use of prednisolone > 7.5 mg daily and hydroxychloroquine > 400 mg daily was prohibited. See Appendix S3 for full eligibility criteria.

Two active lesions from the same anatomical area per participant were each randomized to GSK2646264, administered topically once daily as a 1% (w/w) strength cream or placebo, both with identical excipients for a period of 28 days; all participants received GSK2646264 and placebo. Both GSK2646264 and placebo were formulated as a white/off‐white aqueous cream, manufactured by Medpharm Guildford and stored in amber glass jars at 2–8°C. The maximum applied GSK2646264 dose at any time point was 10 mg/cm^2^ over 90 cm^2^ (900 mg cream containing GSK2646264 9 mg). One participant received placebo and GSK2646264 at the same lesion and was excluded from biomarker, mRNA and efficacy analyses.

The primary endpoint was the safety of GSK2646264 (incidence of adverse events [AEs] and serious AEs [SAEs], clinical safety laboratory assessments, vital signs, electrocardiograms, physical assessments) and the tolerability of GSK2646264 (skin tolerability test that scored dermal response from 0 [no evidence of irritation] to 7 [strong reaction spreading beyond test site] Appendix S3).

Secondary endpoints were pharmacokinetics parameters, change from baseline in Revised Cutaneous Lupus Erythematosus Disease Area and Severity Index (RCLASI)^10^ composite clinical activity score at Days 14 and 28 (used in this trial to assess individual lesions), and mRNA expression levels of several IFN‐related genes in skin biopsies at Day 28. Exploratory endpoints were change from baseline in levels of IFN protein markers, selected immune cell proteins, pSYK and SYK, and histopathology score in skin biopsies at Day 28. Additional details for secondary and exploratory endpoints and an overview of the statistical methods are presented in Appendix S3.

Skin punch biopsies (4 mm) from involved and uninvolved skin were collected predose and at Day 28 and assessed for mRNA expression and protein levels via microarray and immunohistochemistry, respectively.

The study was reviewed and approved by local research ethics committees prior to commencement and conducted in accordance with International Council on Harmonization of Technical Requirements for Pharmaceuticals for Human Use Good Clinical Practice ethical principles and the Declaration of Helsinki. Written informed consent was obtained from each participant prior to study commencement.

## 4 RESULTS

### 4.1 Participant demographics

Participant (*N* = 11) baseline demographics and characteristics are shown in Table 1.

**Table 1:** Participant demographics and baseline characteristics

Characteristic	Total (*N* = 11)
Female, *n* (%)	9 (82)
Age (years)	
Mean (SD)	54.8 (10.44)
Median (range)	53 (39–68)
BMI (kg/m^2^), mean (SD)	23.9 (3.09)
Height (cm), mean (SD)	165.8 (8.42)
Weight (kg), mean (SD)	65.5 (9.63)
Race, *n* (%)	
African American/African	1 (9)
White/Caucasian/European	10 (91)
CLE type, *n* (%)	
Chronic	6 (55)
Acute	0
Subacute	5 (45)
Concomitant medication^a^, *n* (%)^b^	
Any	10 (91)
Hydroxychloroquine sulphate	9 (82)
Prednisolone	5 (45)
Cholecalciferol	3 (27)
Metamizole sodium	3 (27)

Abbreviations: BMI, body mass index; CLE, chronic lupus erythematosus; SD, standard deviation.

^a^ Eligible participants were stable on either no treatment or treatment with: corticosteroids (≤7.5 mg/day prednisone or prednisone equivalent or less) for a minimum of 30 days prior to screening and through to Day 28; hydroxychloroquine (≤400 mg daily dose) for a minimum of 60 days prior to the randomization visit through to Day 28; topical steroids applied to areas of the body that are not exposed to GSK2646264 from screening to Day 28; topical calcineurin inhibitors and retinoids applied to areas of the body that are not exposed to GSK2646264 from screening to Day 28; opioids, if required for acute and chronic pain management.

^b^ Only medications being taken by three or more participants are shown.

### 4.2 Safety and tolerability

GSK2646264 was generally well tolerated; 8/11 (72.7%) participants reported an AE, most commonly nasopharyngitis (3/11; 27.3%). However, only two AEs (headache and hot flush), each reported as one episode in 1/11 (9.1%) participants, were considered treatment‐related; both events resolved. All AEs were mild in intensity, there were no deaths and one SAE was reported (ankle fracture; not treatment‐related).

The skin tolerability test (Appendix S3) was performed predose and up to 1‐h postdose; findings were similar between GSK2646264 and placebo. A maximum dermal response score of 2 was the most frequent score with GSK2646264 (*n* = 8/11; 72.7%) and placebo (*n* = 9/11; 81.8%). Appendix S4 provides additional safety data.

### 4.3 RCLASI score

No difference was observed at any time point for mean overall or modified RCLASI scores between lesions treated with GSK2646264 and placebo (Figure S2), perhaps due to relatively low baseline disease activity.

### 4.4 Plasma pharmacokinetics

Median (range) maximum concentration (C_max_) and time to C_max_ were 1.16 ng/ml (0.1–3.1) and 13.0 days (0.2–27.2), respectively. GSK2646264 concentration remained constant between Days 14 and 28 (Figure S3). These data indicate low systemic exposure to GSK2646264 and were consistent with the first‐in‐human study (clinicaltrials.gov NCT02424799).

### 4.5 Pharmacodynamics and biomarkers

#### 4.5.1 mRNA expression

**FIGURE 1:**
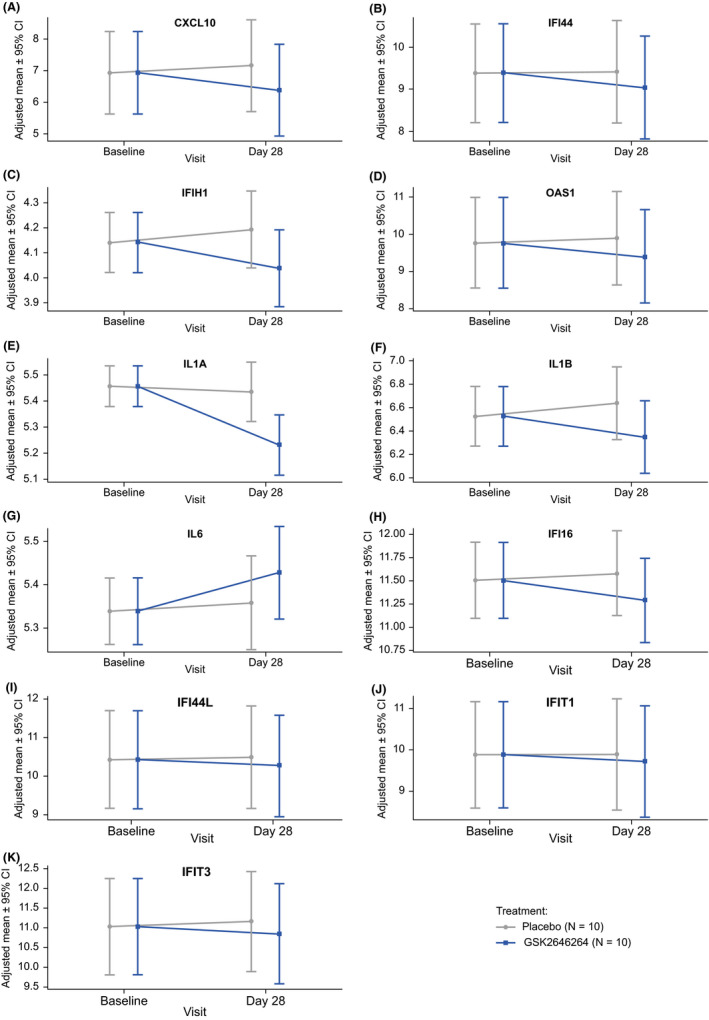
Adjusted mean (95% CI) intensity of log2 mRNA expression by visit and treatment (A) CXCL10, (B) IFI44, (C) IFIH1, (D) OAS1, (E) IL1A, (F) IL1B, and (G) IL6, (H) IFI16, (I) IFI44L, (J) IFIT1 and (K) IFIT3. For genes that had more than one probe analysed, the probe that showed the largest treatment difference is shown. Adjusted mean intensity values were derived using a mixed model with participant as a random effect and treatment as a fixed effect where treatment is set to “not applicable” at baseline. CI, confidence interval

Numerical reductions from baseline in mRNA expression of CXCL10, IFI44, IFIH1 and OAS1 were observed at Day 28 with GSK2646264 vs. placebo (Figure 1A–D), suggesting that the genetic signature associated with CLE may be modulated by GSK2646264. This putative influence on the IFN pathway suggests that targeting of SYK might be beneficial in other IFN‐associated autoimmune diseases, such as Sjogren's syndrome.^11^ However, it is important to interpret the mRNA expression data in light of the small sample size and large confidence intervals, and further studies are required.

The reductions from baseline in IFN‐related gene expression were numerically greater in chronic than subacute CLE. This may be due to a higher IFN‐signature and higher levels of inflammation in participants with chronic vs. subacute CLE.^12^

At Day 28, mean mRNA expression of IL1A and IL1B was reduced from baseline with GSK2646264 compared with placebo at Day 28 (Figure 1E, F). Conversely, IL6 mRNA expression increased from baseline with GSK2646264 vs. placebo at Day 28 (Figure 1G). This was unexpected given that IL6 is an established target of the SYK‐dependent signalling pathway.^13^ Further studies on the influence of GSK2646264 on the SYK pathway may help to identify causes of this discrepancy.

IFI16, IFI44L, IFIT1 and IFIT3 showed decreased expression from baseline after 28 days of GSK2646264, although treatment group differences were minimal (Figure 1H–K). Heatmap representations of the mRNA expression levels of all genes of interest for each participant are shown in Figure S4.

#### 4.5.2 Immune cell marker expression

**FIGURE 2:**
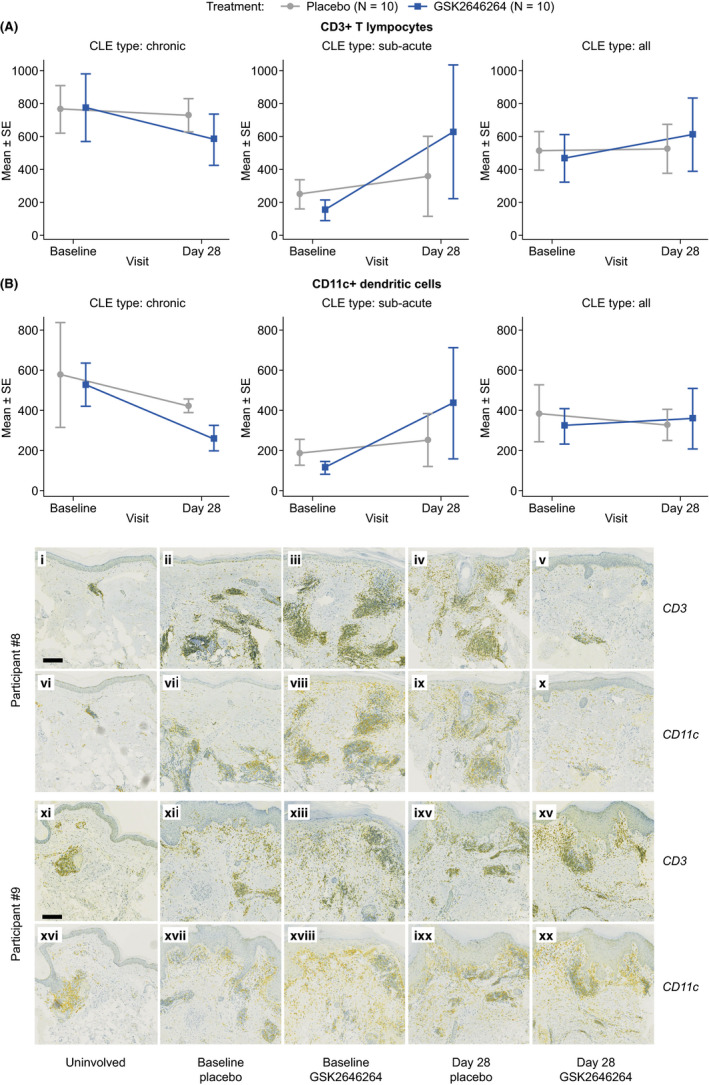
Mean (±SE) expression (cells/mm^2^) of (A) CD3 + T cells and (B) CD11c + dendritic cells in the dermis from biopsies by visit, treatment and sub‐acute/chronic CLE subtypes. Immunohistochemical staining (C) of CD3 + T cells (i–v, xi–xv) and CD11C + dendritic cells (vi–x, xvi–xx) in CLE skin samples in the presence and absence of GSK2646264 treatment from two representative participants. Images represent uninvolved (i, vi, xi, xvi), baseline placebo (ii, vii, xii, xvii), baseline GSK2646264 (iii, viii, xiii, xviii), placebo‐treated at Day 28 (iv, ix, ixv, ixx) and GSK2646264‐treated (x, v, xv, xx) at Day 28. Yellow staining = CD3/CD11c; blue staining = haematoxylin; green = overlap of yellow and blue staining. Scale bars denote 200 µm. Images from Participant #8 and Participant #9 are included as representative images for participants in which immunohistochemical staining changes were and were not observed, respectively. CLE, chronic lupus erythematosus; SE, standard error

A small reduction from baseline in CD3 + T cells and CD11c + dendritic cells in the dermis was observed with GSK2646264 vs. placebo on Day 28 for participants with chronic, but not subacute CLE (Figure 2A, B). These findings are consistent with a previous study, which reported reductions of CD11c + dendritic cells from the skin of MRL‐*lpr* mice with the SYK inhibitor R788.^14^ This may be explained by the involvement of CD3 + T cells and CD11c + dendritic cells in the development of the interface dermatitis at the dermal/epidermal junctional lesions in chronic CLE via production of IFN‐α and IFN‐β.^15^, ^16^ Representative immunohistochemistry images of CD3 + T cell and CD11c + dendritic cell staining from two participants with chronic CLE are shown in Figure 2C.

No changes from baseline in the expression of CD68, CD20 or CD123 were observed in either CLE subtype. Similarly, no changes were observed in expression of pSYK or total SYK; however, assaying phosphoprotein levels within tissue biopsies is challenging due to the dynamic nature of protein phosphorylation and the invasive nature of biopsy.^17^

#### 4.5.3 Histopathology score

No differences were observed between GSK2646264‐ and placebo‐treated lesions in overall histopathology score (Figure S5A) or any of its components. For the dermal inflammation component, there was a modest reduction from baseline to Day 28 with GSK2646264 vs. placebo for the chronic CLE subgroup, but not the subacute subgroup (Figure S5B).

## 5 CONCLUSIONS AND PERSPECTIVES

Topical application of the SYK inhibitor GSK2646264 to active chronic and subacute CLE lesions was well tolerated over 28 days of treatment and no new safety concerns were identified. There was no difference between GSK2646264 and placebo groups in change from baseline in RCLASI, nor in expression of pSYK or total SYK. However, small reductions in the expression of several IFN‐ and inflammatory‐induced genes were observed with GSK2646264 vs. placebo. In participants with chronic CLE, there was a modest reduction in dermal inflammation and levels of CD3 + T cells and CD11c + dendritic cells in the dermis with GSK2646264 vs. placebo.

A key limitation of this study is the small sample size resulting from lower than intended recruitment levels, which precluded a formal statistical analysis of several endpoints; these data should therefore be interpreted with caution.

In conclusion, these putative reductions in the expression of IFN pathway and other inflammatory‐related genes and in dermal inflammation following GSK2646264 treatment in participants with chronic CLE indicate that further studies assessing the efficacy and safety of SYK inhibitors in CLE are warranted.

## CONFLICT OF INTEREST

AW, KF, KN, NW, NR, BH, TSM, SG and HC are employees of GSK and hold stocks/shares in GSK. LE and MD were employees of GSK at the time of the study. JW reports grants from GSK, grants from Incyte, personal fees from Biogen, personal fees from Leo Pharma and other from Novartis. MW received honoraria for consulting by ALK‐Abelló Arzneimittel GmbH, Mylan Germany GmbH, Bencard Allergie GmbH, Novartis AG, Biotest AG, Actelion Pharmaceuticals Deutschland GmbH, Sanofi‐Aventis Deutschland GmbH and HAL Allergie GmbH.

## AUTHOR CONTRIBUTIONS

AW, LE, KF, KN, JW, MW, NW, NR, BH, TSM, SG, HC and MD contributed to the conception or design of the study. JW and MW were involved in the acquisition of the study data. AW, MD, NR, SG and BH contributed to the analysis and interpretation of the data. All authors have read and approved the final manuscript.

## ETHICAL APPROVAL

This study was reviewed and approved by local research ethics committees prior to commencement. The study was conducted in accordance with International Council on Harmonization of Technical Requirements for Pharmaceuticals for Human Use Good Clinical Practice ethical principles and the Declaration of Helsinki. Written informed consent was obtained from each participant prior to study commencement.

## DATA SHARING STATEMENT

Anonymized individual participant data and study documents can be requested for further research from www.clinicalstudydatarequest.com.

## Supporting information


**Appendix S1.** Background
**Appendix S2.** Photoprovocation cohort summary
**Appendix S3.** Supplementary methods
**Appendix S4.** Supplementary results: Safety and tolerability
**Figure S1.** Participant disposition
**Figure S2.** Mean (±SE) (A) overall and (B) modified RCLASI activity scores by visit, treatment and sub‐acute/chronic CLE subtypes
**Figure S3.** Median (range) GSK2646264 plasma concentrations throughout the study
**Figure S4.** Heatmap of log2 mRNA expression levels for gene probes of interest for individual participants at each visit and treatment, shown by sub‐acute and chronic CLE subtypes
**Figure S5.** Mean (±SE) (A) overall histopathology score and (B) histopathology score for the dermal inflammation component by visit, treatment and sub‐acute/chronic CLE subtypes
