# Supplementary material for: Safety, pharmacokinetics and pharmacodynamics of a topical SYK inhibitor in cutaneous lupus erythematosus: A double‐blind Phase Ib study
Source: Exp Dermatol. 2020 Dec 17;30(11):1686–92. doi: 10.1111/exd.14253 (PMC8596667; doi:10.1111/exd.14253)
Supplement: Supplementary file 1 — Appendix S1. Background Appendix S2. Photoprovocation cohort summary Appendix S3. Supplementary methods Appendix S4. Supplementary results: Safety and tolerability Figure S1. Participant disposition Figure S2. Mean (±SE) (A) overall and (B) modified RCLASI activity scores by visit, treatment and sub‐acute/chronic CLE subtypes Figure S3. Median (range) GSK2646264 plasma concentrations throughout the study Figure S4. Heatmap of log2 mRNA expression levels for gene probes of interest for individual participants at each visit and treatment, shown by sub‐acute and chronic CLE subtypes Figure S5. Mean (±SE) (A) overall histopathology score and (B) histopathology score for the dermal inflammation component by visit, treatment and sub‐acute/chronic CLE subtypes [file EXD-30-1686-s001.docx]

# SUPPORTING INFORMATION

## Appendix S1: Background

The inflammation present in cutaneous lupus erythematosus (CLE) lesions is thought to be driven through activation of the interferon (IFN) pathway and potentially mediated by spleen tyrosine kinase (SYK), which is central to several pathways in the immune system, including B-cell maturation and cytokine production.^1^ In a study of 30 patients with CLE, the expression of five Type I IFN-signature marker genes (LYE6E, OAS1, OASL, ISG15 and MX1 was significantly increased in patients with CLE compared with healthy controls; expression of these markers correlated with disease activity.^2^

T cells in patients with systemic lupus erythematosus are known to express abnormally high levels of SYK, resulting in a rewired signaling pathway and transcriptional dysregulation.^3, 4^ ^2^ The active, phosphorylated form of SYK (pSYK) was shown to be strongly expressed in skin lesions of patients with CLE; pSYK-expressing cells were shown to be mostly CD11c+ dendritic cells and T cells.^5^ The expression of several SYK-regulated genes was also upregulated in CLE skin lesions (OAS2, MxA, CXCL9, CXCL10, CCL5, BLNK, PLCG2 and IL21).^5^ Following SYK inhibition in keratinocytes, expression of the IFN marker OAS2 and the pro-inflammatory cytokines CXCL9 and CXCL10 were significantly reduced.^5^

The novel small molecule SYK inhibitor GSK2646264 has demonstrated high inhibitory potency, with a SYK pIC_50_ of 7.1, and selectivity for SYK >316-fold higher than for the key liability target Aurora B.^6^ GSK2646264-mediated inhibition of the SYK downstream target ERK^7, 8^ has also been shown.^6^ In an ex vivo study in human skin, GSK2646264 at concentrations ≥200 ng/mL provided significant inhibition of anti-IgE–induced histamine release from mast cells.^9^ The skin permeability of GSK2646264 supports its use as a topical treatment; 24 hours after application of 1% GSK2646264 cream to intact human skin, the mean dermal concentration was 62.9 µM.^9^

## Appendix S2: Photoprovocation cohort summary

A separate enrolment for participants with 0 or 1 active lesions (‘photoprovocation cohort’) was planned. Participants in the photoprovocation cohort were to undergo photo-testing and daily ultraviolet (UV) photoprovocation on their back for up to 3 consecutive days, providing the opportunity to study the effect of GSK2646264 on non-scarred, non-chronic, and therefore more homogenous lesions. Participants who developed lesions within 14 days of photoprovocation treatment were to undergo treatment with 1% GSK2646264 or placebo for 28 days on separate lesions, with biopsies taken pre- and post-dosing from lesions and uninvolved skin. Two participants were enrolled to the photoprovocation cohort; one participant showed an excessive reaction to photoprovocation and was withdrawn from the study and the other enrolled participant did not receive photoprovocation. Therefore, no participants were randomized to treatment in this cohort, and consequently, the safety and tolerability of GSK2646264 on non-scarred and non-chronic lesions was not assessed. Furthermore, evaluation of GSK2646264 skin concentrations could not be undertaken and the efficacy of GSK2646264 on non-scarred, non-chronic lesions could not be investigated. This represents a potential avenue for future research.

### Photoprovocation model

At screening, UV threshold testing with UVA (≤100 Jcm^-2^) and UVB (≤200 mJcm^-2^) irradiation was performed on six 4.5 cm^2^ areas of the lower back to identify the minimal tanning dose (MTD) for UVA and the minimal erythema dose (MED) for UVB. Three 35 cm^2^ areas of uninvolved skin were planned to be subsequently irradiated daily for three consecutive days with the MTD of UVA followed by 1.5 times the MED of UVB.

## Appendix S3: Supplementary methods

### Eligibility criteria

Participants 18–70 years of age with a confirmed diagnosis of subacute or chronic CLE who met the following criteria were included: thyroid-stimulating hormone, free T4 and free T3 values within the normal range; body mass index ≥19 kg/m^2^; male, or females of non-reproductive potential (or of reproductive potential and agreed to use a highly effective method of contraception); free from scarring, skin markings or wounds in the areas of the body to be treated and able to refrain from extended direct sunlight and tanning products on the areas to be treated. Subacute and chronic CLE subgroups diagnoses were obtained at assessment and confirmed through histological assessment.

Participants were excluded if they had: alanine aminotransferase values more than twice the upper limit of normal (ULN); bilirubin more than 1.5 times the ULN; current or chronic history of liver disease or known hepatic or biliary abnormalities; QTcF>450 msec; a history of past or present benign or malignant skin conditions and disease; active SLE and/or significant disease in any other organ than the skin; a history of Graves’ disease or thyroid cancer; use of any prohibited medications during the study (prednisolone >7.5 mg daily or any increase in dose from screening to Day 28; hydroxychloroquine >400 mg daily or any increase in dose from screening to Day 28; photosensitizing drugs within 5 half-lives before photoprovocation); direct exposure of the testing areas to UV light within 2 weeks of study entry. Participants in the photoprovocation cohort were withdrawn if they experienced an excessive or adverse reaction to the photoprovocation.

### Secondary and exploratory objectives: Additional information

- **Revised Cutaneous Lupus Erythematosus Disease Area and Severity Index (RCLASI):** Overall and modified scores were assessed. The modified RCLASI is derived by summing scores for erythema, scaling/hyperkeratosis and edema/infiltration.
- **mRNA expression levels via microarray**: Included expression of several IFN-related genes, including CXCL10, IFI16, IFIH1, OAS1, IL1A, IL1B, IL6, IFI44, IFI44L, IFIT1 and IFIT3.
- **Protein levels via immunohistochemistry:** Performed according to the skin area (in dermis, epidermis and total area biopsies). Included assessment of IFN protein marker (MxA), selected immune cell proteins (CD3, CD11c, CD68, CD20 and CD123), pSYK and SYK.
- **Histopathology score**: based on pathological evaluation of biopsies to assess parakeratosis, ballooning, necrosis, junctional inflammation and the extent of dermal infiltrate.

### Skin tolerability test

Local tolerability was assessed with the following skin irritation scoring system, where the

score consists of a numeric score according to the dermal response scoring, and a letter

according to other effects.

- **Dermal response scoring:**

0 = no evidence of irritation

1 = minimal erythema, barely perceptible (pink)

2 = moderate erythema (definite redness), readily visible; minimal edema or minimal papular response

3 = strong erythema (intense redness), or erythema and papules

4 = definite edema

5 = erythema, edema and papules

6 = vesicular eruption

7 = strong reaction spreading beyond test site

- **Other effects:**

Z = no other effect

A = slight glazed appearance

B = marked glazing

C = glazing with peeling and cracking

F = glazing with fissures

G = film of dried serous exudate covering all or part of the dose site

H = small petechial erosions and/or scabs

### mRNA expression

RNA was prepared from one 4 mm biopsy that had been cut in half, perpendicular to the surface of the skin. The subcutaneous fat was trimmed from the biopsy to reduce contamination of the transcriptome with the adipose layer of skin. It is acknowledged that there was a mixed cell population within each biopsy, and variability in skin thickness and the level of inflammation. To minimize variability, biopsies were taken from representative active areas of the lesions, with repeat biopsies at Day 28 taken from the same active area >1 cm from the original biopsy site.

A TRIzol-chloroform extraction method was used for mRNA preparation and RNA concentration was quantified by measuring UV absorbance at 260 nm using a NanoDrop 8000 spectrophotometer (ThermoFisher Scientific, Waltham, USA). RNA quality was assessed using RNA 6000 Nano chips on an Agilent 2100 Bioanalyser (Agilent Technologies, Santa Clara, USA). A minimum RNA integrity number (RIN) of 6.4 was required for inclusion in subsequent analyses. Samples with an acceptable RIN were used for cDNA amplification and for analysis using Affymetrix U133 Plus 2.0 microarray chips (ThermoFisher Scientific, Waltham, USA), with data normalized in Array Studio v5.0 (OmicSoft, Cary, USA).

### Immunohistochemistry

Immunohistochemistry biopsy samples were collected in formalin prior to embedding in paraffin and 3.5 μm sections were obtained. Antibodies were used to detect CD3 (clone 2GV6, Ventana Medical Systems, Oro Valley, USA), CD11c (clone 5D11, Cell Marque, Rocklin, USA), CD20 (clone L26, Ventana Medical Systems, Oro Valley, USA), CD68 (clone KP1, Ventana Medical Systems, Oro Valley, USA), CD123 (clone 7G3, BD Biosciences, San Jose, USA), MXA (clone M143, EMD Millipore, Burlington, USA), pSYK (clone C87C1, Tyr535/536, Cell Signalling Technology, Danvers, USA) and SYK (clone SYK-01, BioLegend, San Diego, USA). The Ventana Discovery Ultra Autostainer with Ventana Discovery secondary detection reagents were used for staining. Images were acquired using a Nanozoomer slide scanner (Hamamatsu Photonics, Hamamatsu, Japan) and analyzed within HALO image analysis software (Indica Labs, Albuquerque, USA). An automated image analysis algorithm was used to detect epidermis, dermis and total skin regions of interest and cells classified positive for the immuno-label were numerated within these regions, overseen by a manual quality control process.

### Statistical analysis

A total of 40 participants were planned to be recruited into the study; 30 participants for the active lesion cohort and 10 participants for the photoprovocation cohort; this was based on feasibility, primarily to allow for the assessment of safety and tolerability. However, after the start of the study, due to recruitment issues, the target number for the active lesion and photoprovocation cohorts were revised down to 15 and 5 participants, respectively. Safety data were summarized descriptively. Plasma GSK2646264 concentrations over time were summarized and presented graphically and derived pharmacokinetics parameters were summarized. Descriptive summaries of pharmacodynamic data were produced by CLE subtype to allow for comparisons between participants diagnosed with chronic and subacute CLE. Descriptive summaries and listings were produced for RCLASI scores; however, due to the small final number of participants recruited, no formal statistical analysis could be performed for RCLASI scores as planned. Change from baseline for IFN and inflammatory marker mRNA levels were analyzed using a mixed effects model, with treatment and lesion number as fixed effects and participant as a random effect, in order to estimate treatment means by visit and fold changes from baseline to Day 28.

## Appendix S4: Supplementary results: Safety and tolerability

In addition to the results reported in the main manuscript text, other adverse events reported by participants were: tinea pedis, post-procedural or post-operative wound, headache, migraine, contact dermatitis, panniculitis, pruritis, constipation, diarrhea, back pain and hot flush (all n=1/11; 9.1%).

One participant recorded a maximum dermal response score of 3 (strong erythema or erythema and papules) during the 29- to 42-day period following treatment with GSK2646264, whereas no participant recorded a score of 3 when treated with placebo. One participant, when treated with placebo, recorded a dermal score of 2 (moderate erythema, readily visible; minimal edema or minimal papular response), in addition to marked glazing over the first 14 days of dosing, which resolved by the end of the study.

## Supplementary Figure S1: Participant disposition


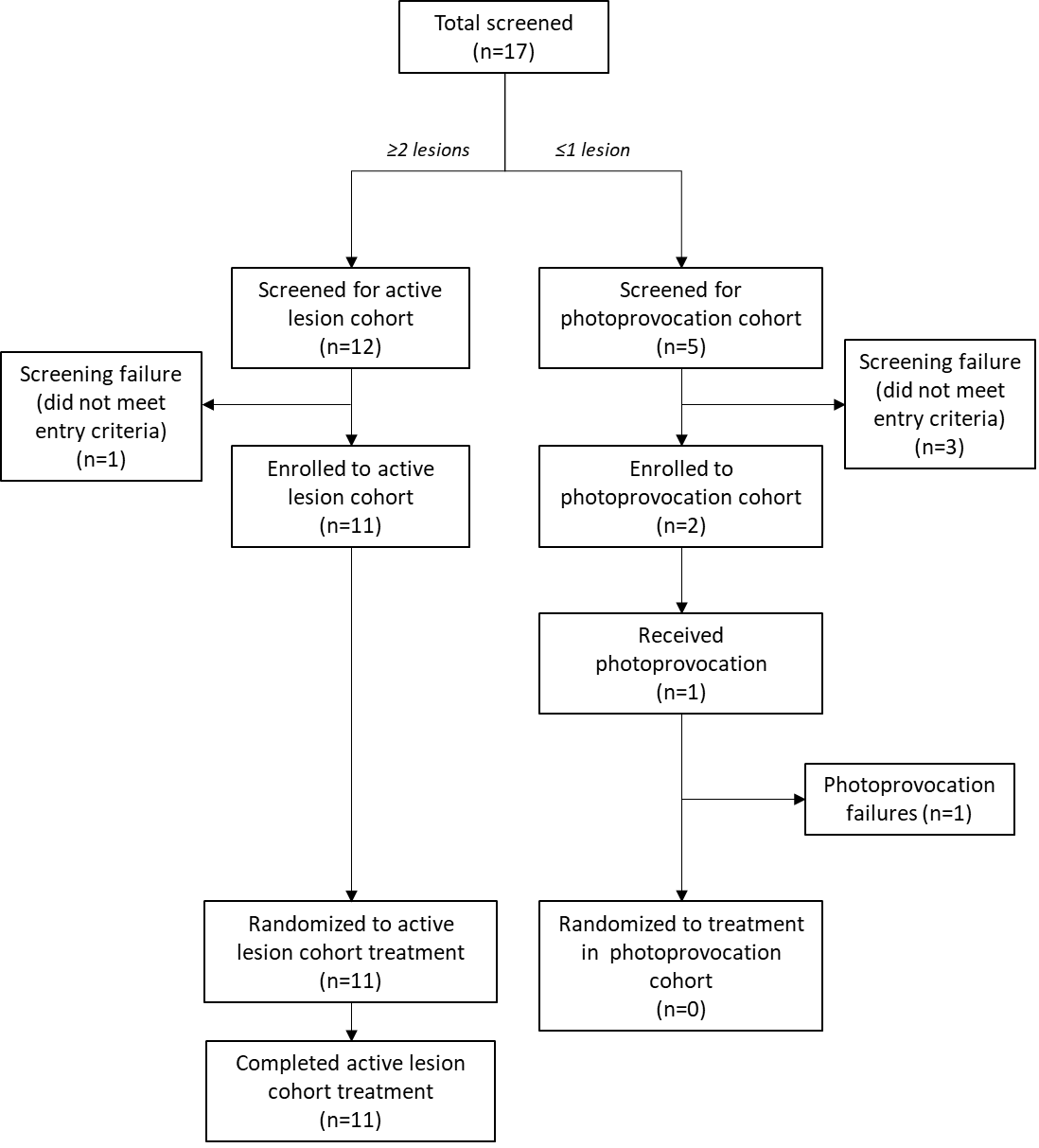


A ‘completed’ participant had received at least 25 doses of study treatment and completed to the end of treatment biopsy and assessment at Day 28.

One participant in the photoprovocation cohort was enrolled but did not receive photoprovocation due to the participant’s availability.

## Supplementary Figure S2: Mean (±SE) (A) overall and (B) modified RCLASI activity scores by visit, treatment and sub-acute/chronic CLE subtypes


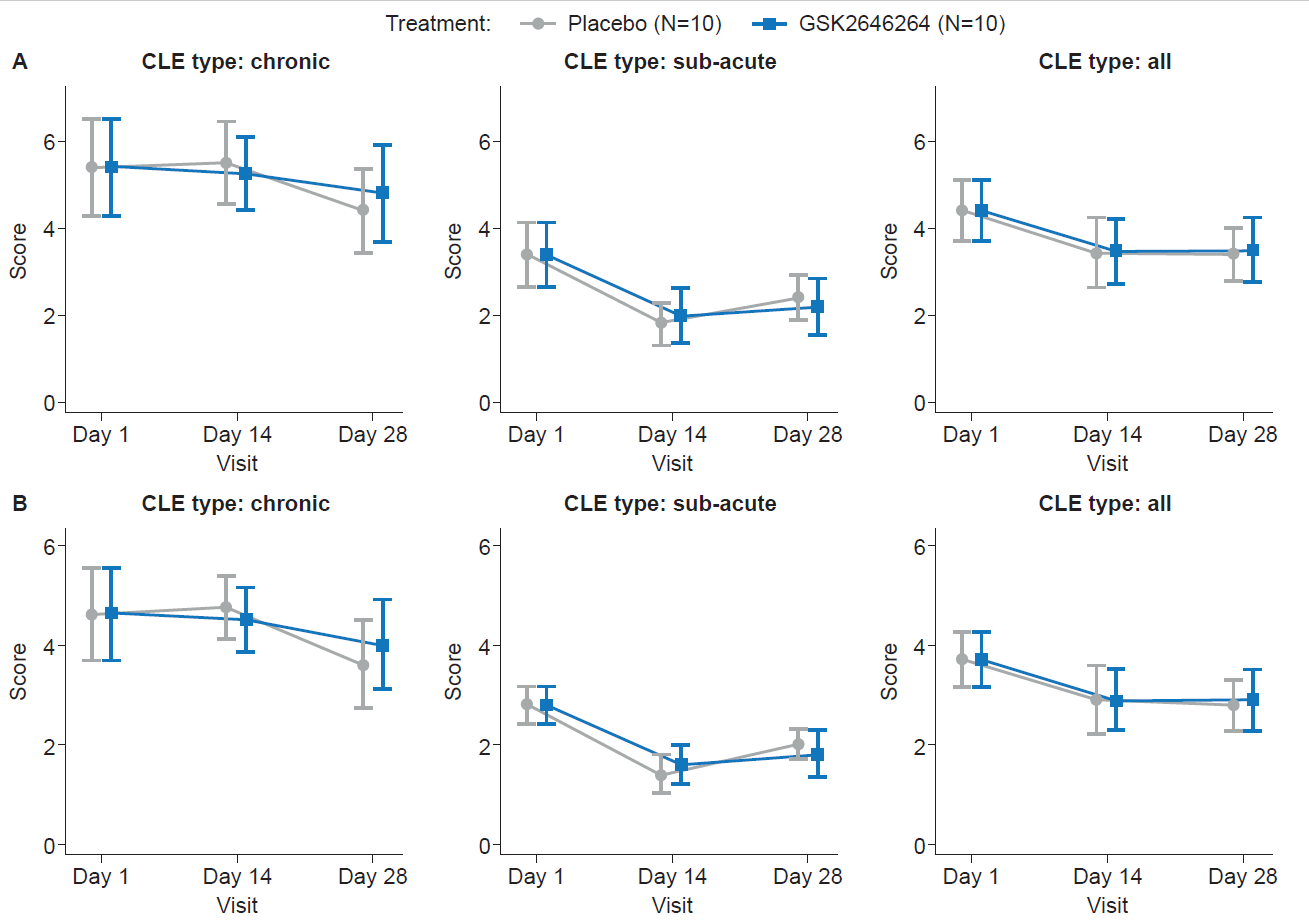


CLE, chronic lupus erythematosus; RCLASI, Revised Cutaneous Lupus Erythematosus Disease Area and Severity Index; SE, standard error.

## Supplementary Figure S3: Median (range) GSK2646264 plasma concentrations throughout the study


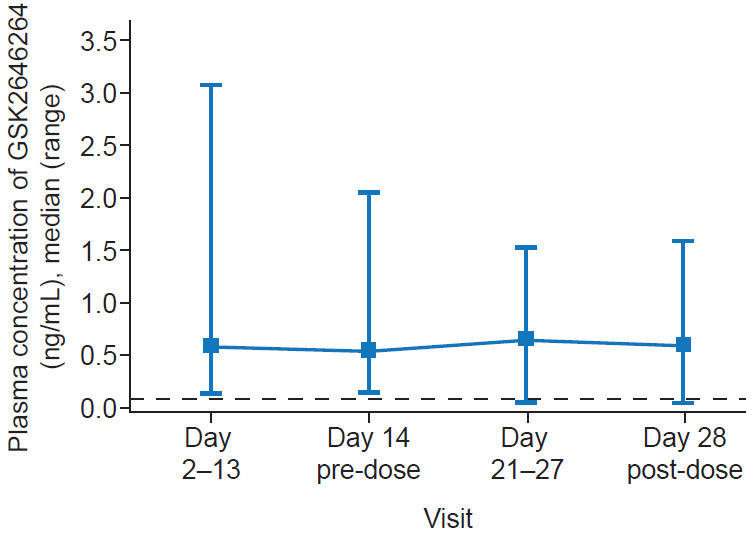


One value at each of the Day 21–27 and Day 28 post-dose visits were imputed. If more than 30% of concentration values are imputed, then the median (range) are not displayed. The dotted line represents the lower limit of quantification (LLQ) (0.05 ng/mL).

## Supplementary Figure S4: Heatmap of log2 mRNA expression levels for gene probes of interest for individual participants at each visit and treatment, shown by sub-acute and chronic CLE subtypes


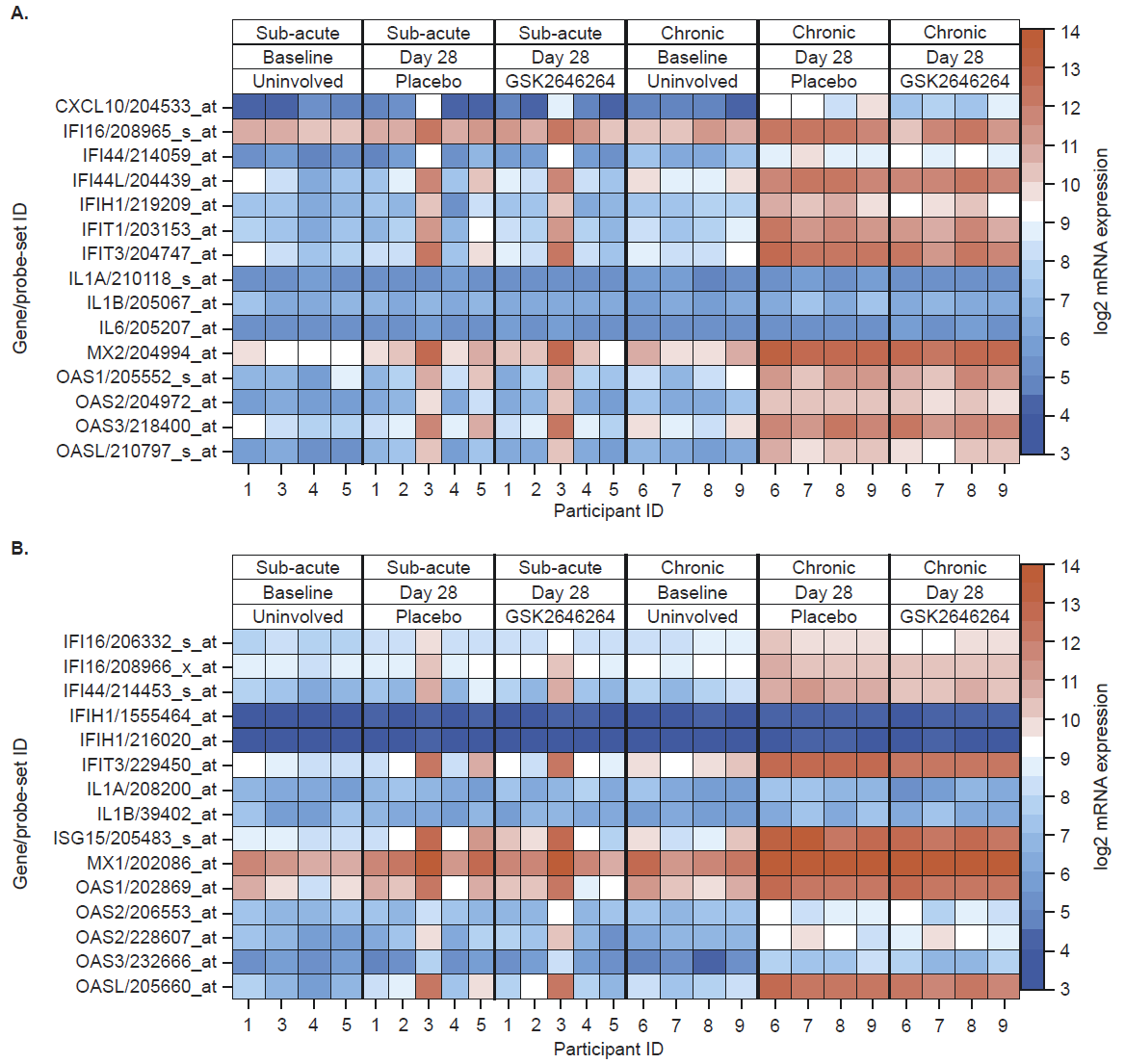


## Supplementary Figure S5: Mean (±SE) (A) overall histopathology score and (B) histopathology score for the dermal inflammation component by visit, treatment and sub-acute/chronic CLE subtypes


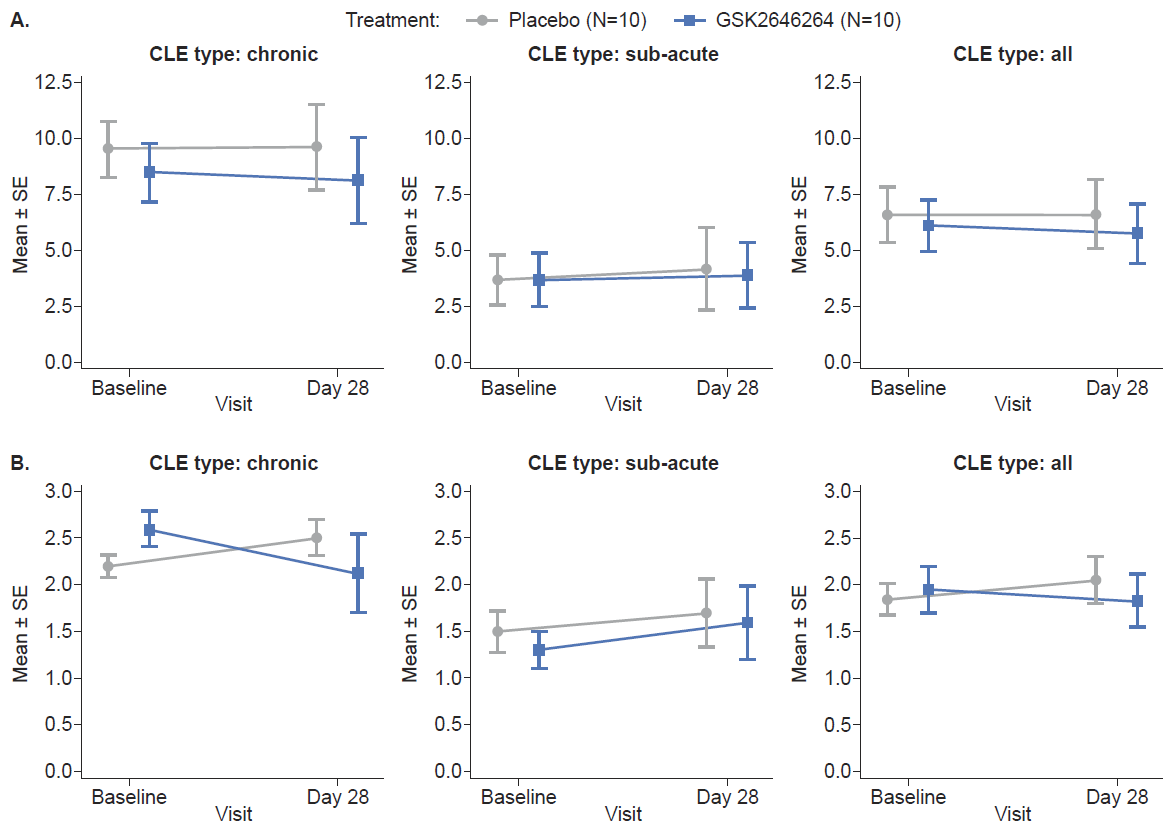


CLE, cutaneous lupus erythematosus; SE, standard error.

**REFERENCES**

1. Mócsai A, Ruland J, Tybulewicz VLJ. The SYK tyrosine kinase: a crucial player in diverse biological functions. Nat Rev Immunol. 2010;10:387-402. doi:10.1038/nri2765.

2. Braunstein I, Klein R, Okawa J, Werth VP. The interferon-regulated gene signature is elevated in subacute cutaneous lupus erythematosus and discoid lupus erythematosus and correlates with the cutaneous lupus area and severity index score. Br J Dermatol. 2012;166:971-975. doi:10.1111/j.1365-2133.2012.10825.x.

3. Grammatikos AP, Ghosh D, Devlin A, Kyttaris VC, Tsokos GC. Spleen Tyrosine Kinase (Syk) Regulates Systemic Lupus Erythematosus (SLE) T Cell Signaling. PLoS One. 2013;8:e74550. doi:10.1371/journal.pone.0074550.

4. Liossis SN, Ding XZ, Dennis GJ, Tsokos GC. Altered pattern of TCR/CD3-mediated protein-tyrosyl phosphorylation in T cells from patients with systemic lupus erythematosus. Deficient expression of the T cell receptor zeta chain. J Clin Invest. 1998;101:1448-1457. doi:10.1172/jci1457.

5. Braegelmann C, Holzel M, Ludbrook V, et al. Spleen tyrosine kinase (SYK) is a potential target for the treatment of cutaneous lupus erythematosus patients. Exp Dermatol. 2016;25:375-379. doi:10.1111/exd.12986.

6. Barker MD, Liddle J, Atkinson FL, et al. Discovery of potent and selective Spleen Tyrosine Kinase inhibitors for the topical treatment of inflammatory skin disease. Bioorg Med Chem Lett. 2018;28:3458-3462. doi:10.1016/j.bmcl.2018.09.022.

7. Gurung P, Fan G, Lukens JR, Vogel P, Tonks NK, Kanneganti TD. Tyrosine Kinase SYK Licenses MyD88 Adaptor Protein to Instigate IL-1alpha-Mediated Inflammatory Disease. Immunity. 2017;46:635-648. doi:10.1016/j.immuni.2017.03.014.

8. Slack EC, Robinson MJ, Hernanz-Falcon P, et al. Syk-dependent ERK activation regulates IL-2 and IL-10 production by DC stimulated with zymosan. Eur J Immunol. 2007;37:1600-1612. doi:10.1002/eji.200636830.

9. Ramirez Molina C, Falkencrone S, Skov PS, Hooper-Greenhill E, Barker M, Dickson MC. GSK2646264, a spleen tyrosine kinase inhibitor, attenuates the release of histamine in ex vivo human skin. Br J Pharmacol. 2019;176:1135-1142. doi:10.1111/bph.14610.
